# A Novel *ARL3* Gene Mutation Associated With Autosomal Dominant Retinal Degeneration

**DOI:** 10.3389/fcell.2021.720782

**Published:** 2021-08-17

**Authors:** Rinki Ratnapriya, Samuel G. Jacobson, Artur V. Cideciyan, Milton A. English, Alejandro J. Roman, Alexander Sumaroka, Rebecca Sheplock, Anand Swaroop

**Affiliations:** ^1^Neurobiology-Neurodegeneration and Repair Laboratory, National Eye Institute, National Institutes of Health, Bethesda, MD, United States; ^2^Department of Ophthalmology, Baylor College of Medicine, Houston, TX, United States; ^3^Department of Ophthalmology, Perelman School of Medicine, Scheie Eye Institute, University of Pennsylvania, Philadelphia, PA, United States

**Keywords:** chromatic perimetry, ciliopathy, cones, electroretinography, retinal degeneration, rods, whole exome sequencing

## Abstract

Despite major progress in the discovery of causative genes, many individuals and families with inherited retinal degenerations (IRDs) remain without a molecular diagnosis. We applied whole exome sequencing to identify the genetic cause in a family with an autosomal dominant IRD. Eye examinations were performed and affected patients were studied with electroretinography and kinetic and chromatic static perimetry. Sequence variants were analyzed in genes (*n* = 271) associated with IRDs listed on the RetNet database. We applied a stepwise filtering process involving the allele frequency in the control population, *in silico* prediction tools for pathogenicity, and evolutionary conservation to prioritize the potential causal variant(s). Sanger sequencing and segregation analysis were performed on the proband and other family members. The IRD in this family is expressed as a widespread progressive retinal degeneration with maculopathy. A novel heterozygous variant (c.200A > T) was identified in the *ARL3* gene, leading to the substitution of aspartic acid to valine at position 67. The Asp67 residue is evolutionary conserved, and the change p.Asp67Val is predicted to be pathogenic. This variant was segregated in affected members of the family and was absent from an unaffected individual. Two previous reports of a *de novo* missense mutation in the *ARL3* gene, each describing a family with two affected generations, are the only examples to date of autosomal dominant IRD associated with this photoreceptor gene. Our results, identifying a novel pathogenic variant in *ARL3* in a four-generation family with a dominant IRD, augment the evidence that the *ARL3* gene is another cause of non-syndromic retinal degeneration.

## Introduction

Emerging from the era of ungenotyped inherited retinal degenerations (IRDs), we are now aware of the heterogeneous basis of these blinding diseases (Bramall et al., 2010; Wright et al., 2010; Ratnapriya and Swaroop, 2013; Verbakel et al., 2018; Garafalo et al., 2020). From the linkage mapping of disease loci to the identification of causative genes and mutations, there was a steady increase in the number of genes associated with IRDs in the three decades from 1990 onward (RetNet, the Retinal Information Network)^1^. Yet, there remain many IRD patients and families with unknown genetic diagnosis (at least 30%; Birtel et al., 2018; Garafalo et al., 2020; Hejtmancik and Daiger, 2020). The largest percentage of these molecularly unresolved Mendelian IRDs has been the simplex/multiplex or presumed autosomal recessively inherited diseases (Garafalo et al., 2020).

We have been investigating patients and families with non-syndromic retinal degeneration, and whenever a genetic cause for an autosomal dominant IRD was identified, the family was screened for known mutations. In recent years, the next-generation sequencing technologies, especially targeted and whole exome sequencing, have expedited the molecular diagnosis efforts (Booij et al., 2011; O’Sullivan et al., 2012; Ratnapriya and Swaroop, 2013; Beryozkin et al., 2015; Roberts et al., 2016). In the current study, we applied whole exome sequencing to a multi-generation dominant IRD family which was initially screened for known mutations but gave negative results. We analyzed all genes associated with IRDs as reported in the RetNet database and identified a novel, rare, heterozygous variant p.Asp67Val in *ARL3* as a causative mutation. *ARL3* encodes ADP-ribosylation factor, (Arf)-like protein 3. This soluble small GTPase has been localized to photoreceptors, and mutations in the *ARL3* gene are considered to cause retinal ciliopathy (Frederick et al., 2020; Sánchez-Bellver et al., 2021).

A missense variant in *ARL3* has previously been associated with non-syndromic autosomal dominant retinitis pigmentosa (OMIM 604695; Fahim et al., 2000). Specifically, the c.269A > G (p.Tyr90Cys) variant was determined to be a *de novo* mutation in two unrelated families, each with two generations of affected members (Strom et al., 2016; Holtan et al., 2019). *ARL3* has also been implicated as an autosomal recessive cause of Joubert syndrome and non-syndromic retinal degeneration (Alkanderi et al., 2018; Sheikh et al., 2019; Fu et al., 2021).

The identification of causal genes underlying human diseases has clear clinical and research utility, and there has been recent progress toward therapy in dominant forms of IRD (Sudharsan and Beltran, 2019; Kruczek et al., 2021). Further, specifically considering the *ARL3* gene, there are studies in patient-derived cell lines and animal models that can be the foundation for understanding the mechanism and devising the therapeutic strategies (Grayson et al., 2002; Evans et al., 2010; Schwarz et al., 2012; Hanke-Gogokhia et al., 2016; Kruczek and Swaroop, 2020).

## Materials and Methods

### Human Subjects

Patients from a four-generation family with a history of visual impairment were included (Table 1). Six family members representing three of the generations underwent a standard ophthalmic examination as well as specialized psychophysical, electrophysiological, and imaging tests. Their clinical visits occurred between 1983 and 2002; molecular studies to identify the causative gene in this family were performed throughout the period from 1992 to 2021.

**TABLE 1:** Clinical characteristics of *ARL3* family.

**Patient number/Sex**	**Age at first visit (years)**	**Ages at follow-up (years)**	**Best corrected visual acuity (first visit)**		**Refractive error (first visit) as spherical equivalent**	
			**OD**	**OS**	**OD**	**OS**
II-1/M	69	One visit	HM	HM	−0.75	−0.75
III-1/M	47	55	20/300	HM	−3.50	−4.00
III-2/M	41	42, 46, 48, 52	20/20	20/25	−0.50	−1.00
IV-1/F	13	14, 15, 16, 18, 20	20/50	20/40	−9.00	−7.00
IV-2/F	10	12, 15, 18, 21	20/40	20/30	−4.50	−5.00
IV-3/F	8	9, 10, 13, 15, 17, 19, 27	20/30	20/40	−3.25	−2.75

### Phenotype Studies

#### Full Field Electroretinography (ERG)

Techniques, methods of data analysis, and normal results for ERGs have been described (Jacobson et al., 1989; Yagasaki and Jacobson, 1989; Azari et al., 2006; Aleman et al., 2009). After a 45-min period of dark adaptation, full-field ERGs were elicited using scotopically matched blue and red lights followed by a series of blue light flashes with increasing intensities to elicit a rod response. Bright flashes of white light were used to obtain a mixed cone and rod response. To elicit a cone response, single flashes of white light on a steady white background, suppressing the rods, was used. Cone function was also measured by using a series of white light flickering at 30 Hz with increasing intensities on a white background. The relationship of rod b-wave amplitude to cone flicker amplitude was calculated and plotted (Yagasaki and Jacobson, 1989).

To directly assess the function of rod-photoreceptor phototransduction activation and rod-driven post-receptoral signaling in patient III-2 at age 52, photoresponses were elicited with different luminances of blue (Wratten 47A) and red (Wratten 26) flashes in the dark-adapted state as previously described (Cideciyan and Jacobson, 1993, 1996; Cideciyan, 2000). In brief, luminances were selected to provide pairs of scotopically matched waveforms to blue and red flashes which when digitally subtracted gave a cone ERG that was then subtracted from the response to a photopically matched blue flash (double subtraction technique). The resulting rod-isolated ERG was assumed to be the sum of two major underlying components, P3 and P2. P3 is generated by the photoreceptors, and P2 is generated by the inner nuclear layer. The P3 component of the rod-isolated ERG was quantitatively estimated by fitting a physiologically based mathematical model previously developed (Cideciyan and Jacobson, 1996) for the leading edges of the waveforms. Then the rod-driven post-receptoral P2 component was derived by subtracting the P3 estimate from the rod-isolated ERG. The derived P2 component was normalized by the maximum size of the P3 component to facilitate comparison between the different amplitudes of patients and normal subjects.

#### Perimetry

Goldmann kinetic perimetry was performed with V-4e and I-4e test targets. Static perimetry was performed with a modified automated perimeter (Humphrey Field Analyzer 750i) as previously described (Jacobson et al., 1986; Roman et al., 2005). Two-color (500 and 650 nm) dark-adapted function and light-adapted function (600 nm) were measured at 2° intervals across the central visual field (central 60° along horizontal and vertical meridians) and at 12° intervals throughout the visual field. Photoreceptor mediation under dark-adapted conditions was determined by the sensitivity difference between 500- and 650-nm stimuli (Jacobson et al., 1986; Roman et al., 2005).

#### Imaging

Color fundus photographs were obtained using camera systems available at the time of examinations. In one family member, cross-sectional retinal reflectivity profiles were obtained with optical coherence tomography (OCT; Zeiss Humphrey Instruments, Dublin, CA, United States). Our OCT techniques have been published (e.g., Jacobson et al., 2003; Sumaroka et al., 2019). For the current work, data were acquired with first-generation (time-domain) OCT1 with a theoretical axial resolution in retinal tissue of ∼10 μm. Scans were composed of 100 longitudinal reflectivity profiles (LRPs). Five overlapping segments of linear scans (repeated two times each of 4.5-mm length) located along the horizontal and vertical meridian, centered on the anatomical fovea and extending to 9 mm in either direction, were acquired. Post-acquisition processing of OCT data was performed with custom programs (MATLAB R2020b; MathWorks, Natick, MA, United States). LRPs making up the OCT scans were aligned by straightening the major RPE reflection, and scans from the same eccentricities were averaged.

### Molecular Studies

#### Whole Exome Sequencing Analysis

Genomic DNA was extracted from the peripheral blood using standard methods and quantified using the Promega QuantiFluor^®^ dsDNA system (Promega, Madison, WI, United States), according to the manufacturer’s instructions. Targeted exome capture was performed using genomic DNA from the proband using the Agilent SureSelect Human All exon 50 Mb kit (Agilent Technologies, Santa Clara, CA, United States), following the manufacturer’s instructions. Captured libraries were amplified and converted to clusters using Illumina Cluster Station, and paired-end 126 bp sequencing was performed on Illumina GAIIx (Illumina, Inc., San Diego, CA, United States).

#### Primary Bioinformatics Analysis

FastQC (available at http://www.bioinformatics.babraham.ac.uk/projects/fastqc/) was used to confirm the quality of sequencing, and adapter indexes were removed using Trimmomatic (Bolger et al., 2014). Sequence reads were mapped against the hg38/GRCh38 human reference genome sequence build using BWA. Aligned reads were processed to mark duplicates using Picard^2^. The Genome Analysis Toolkit (GATK) recommendations for best practices were applied for variant calling, local realignment, base quality recalibration, and variant recalibration (DePristo et al., 2011). The annotation of variants was performed with ANNOVAR (Wang et al., 2010). As a part of ANNOVAR annotations, alternate allele frequencies of the variants in non-Finnish European (NFE) populations were obtained from the Exome Aggregation Consortium (ExAC), which constituted data from 33,370 individuals.

#### Variant Filtering and Prioritization

**FIGURE 1:**
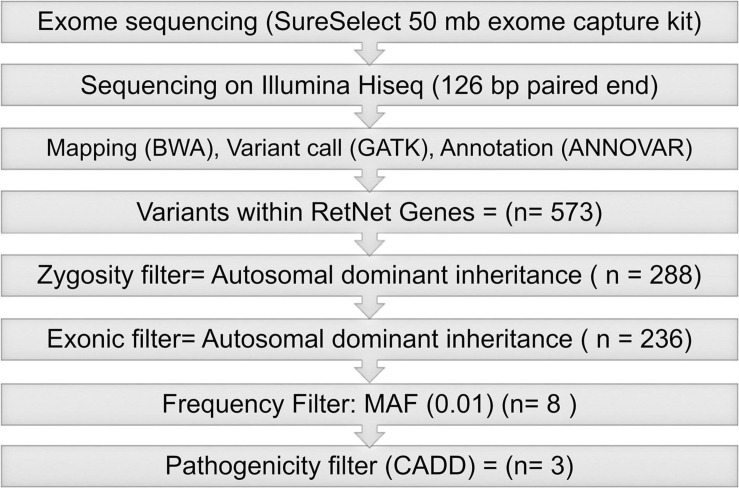
Analysis and filtering pipeline for RetNet genes.

To identify pathogenic variants, we applied a stepwise filtering process on sequence variants identified in genes listed on the RetNet database (in the public domain; accessed July 2020)^3^ (Figure 1). First, we retained only functional coding variants (non-synonymous, stop gain, stop loss, splicing, and frameshift insertions/deletions). Next, we excluded variants with a minor allele frequency (MAF) of >0.01 in the ExAC as they were deemed not disease causing because of their prevalence in the apparently normal population. The variants were subsequently selected based on an additional predictive causality filter to focus on the 1% most deleterious variants in the human genome (CADD score ≥ 20).

#### Confirmation of Candidate Variants by Sanger Sequencing

Candidate variant in *ARL3* identified by whole exome sequencing was validated by Sanger sequencing in the proband and five additional family members (four affected and one unaffected) using an ABI PRISM 3730xl Genetic Analyzer (Applied Biosystems; Thermo Fisher Scientific, Waltham, MA, United States). The primers used to amplify the *ARL3* DNA fragment containing Asp67Val variant were ARL3-F-5′-TCACAACAAATCATTTTCAGCA-3′ and ARL3-R-5′-CTGAGCGACAGCAAAACATC-3′.

#### Conservation of Asp67Val Across Species

We next analyzed the evolutionary conservation of the variant as it is a strong indicator of the deleteriousness of any change. Multiple species protein sequence alignment to assess evolutionary conservation of residue was performed using the UCSC Genome Browser^4^.

#### Protein–Protein Interaction Prediction

To understand the effects of p.Asp67Val mutation, we took advantage of the ARL3 interaction structural information in predicting the impact of this substitution on the affinity of protein–protein complexes. We used mCSM-PPI2 (Rodrigues et al., 2019), which is a machine-learning method that relies on graph-based signatures to evaluate the structure-based prediction of the impact of missense mutations on protein-protein interaction. p.Asp67Val was modeled with mCSM-PPI2 on the structures 3BH6 (RP2), 4GOJ (UNC119A), and 5DI3 (ARL13B).

## Results

### Phenotype Studies

#### Clinical Features of the IRD in This Family

**FIGURE 2:**
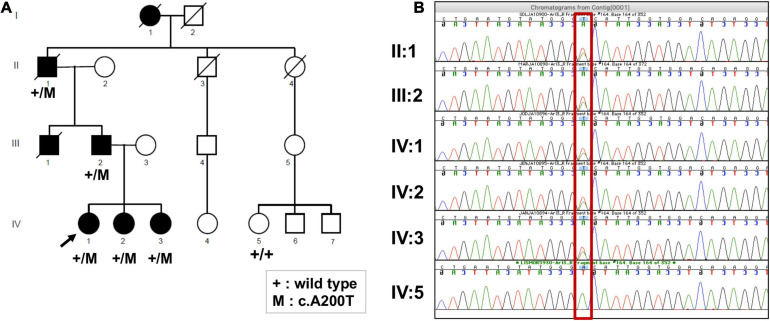
**(A)** Pedigree of the family, showing members affected and unaffected by the IRD. The segregation of the *ARL3* variant c.A200T is shown where mutation is indicated by “+/M,” and its absence is indicated by “+/+.” **(B)** Sanger sequencing electropherogram of the available family members confirming the exome sequencing results, as all affected are heterozygous (A/T) and the unaffected is homozygous for the wild-type allele (A/A) (shown in red rectangle).

The four-generation Ashkenazi-Jewish family with a history of progressive visual loss in some members but no other systemic disease manifestations (Figure 2A and Table 1) was studied to determine the ocular-retinal phenotype. From the examination of the oldest affected member (PII/1, age 69), it was evident that the retinal disease expression could be severe at this age; night vision losses were recalled by the patient in his third decade of life, and then there was progression to peripheral and central vision loss over subsequent decades. Posterior subcapsular cataracts (PSCs) were present at age 69. The fundus appearance was one of widespread pigmentary retinopathy and chorioretinal atrophy as well as waxy disk pallor and attenuated vessels. An ERG was non-detectable; and kinetic visual fields were not measurable. PIII-1 at age 47 years also had a severe disease expression; central and night vision disturbances were present from the second decade of life. Peripheral vision loss prompted mobility training in his early fourth decade. Acuities were 20/300 with hand motions at first examination (Table 1), and only a small central island of visual field was present. ERGs were not detectable at age 47.

In contrast to the severe disease expression of PII-1 and PIII-1, PIII-2 was asymptomatic early in his fifth decade of life. The three daughters of PIII-2 were all examined late in their first or early in their second decades of life. Two (PIV-2 and -3) of the three were asymptomatic while PIV-1 already had complaints of night vision disturbances. Serial visual acuities of five of the affected family members are shown (Supplementary Figure 1).

**FIGURE 3:**
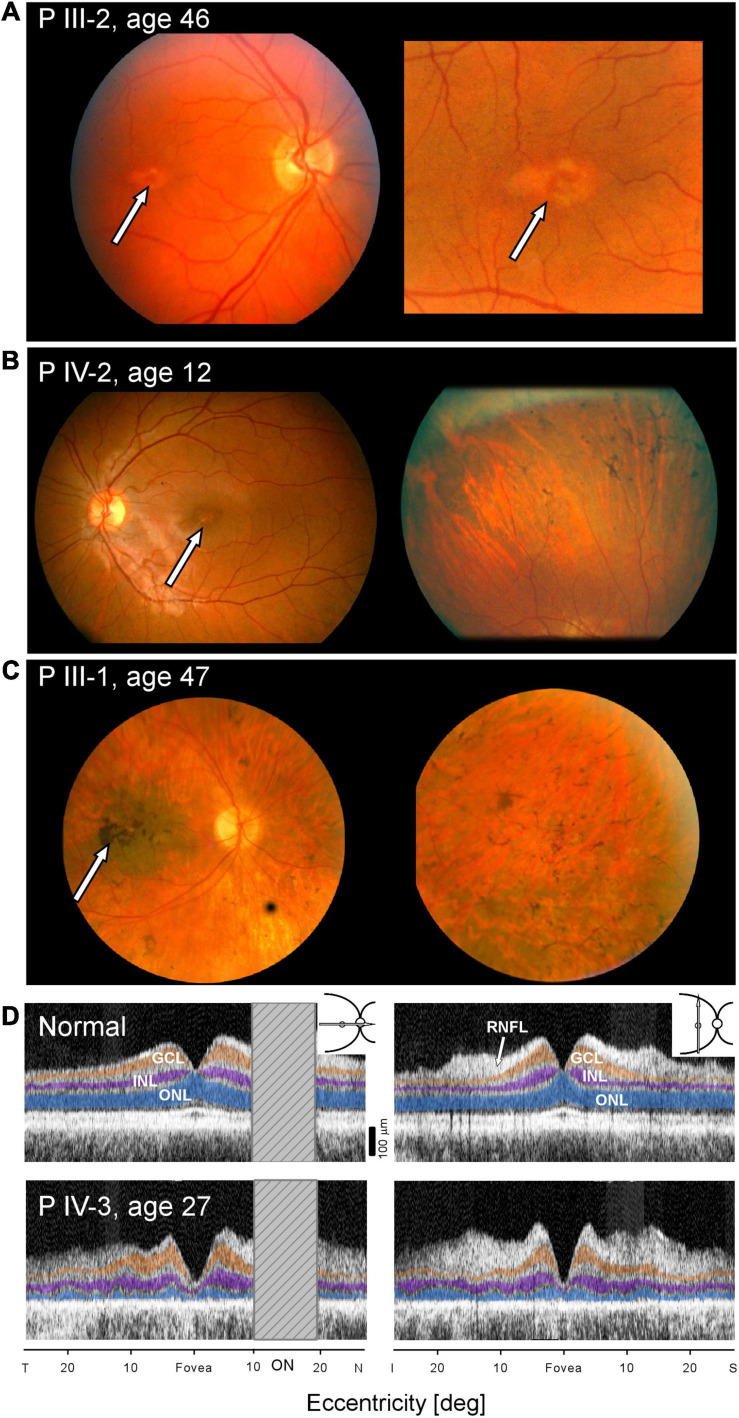
Maculopathy at all stages of the IRD. **(A–C)** Fundus photographs of PIII-2, PIV-2, and PIII-1. Arrows point to maculopathy. **(D)** OCTs of normal subject (upper panels) compared with scans of PIV-3 (lower panels) along horizontal and vertical meridians (insets, upper right). Hatched bar shows the location of the optic nerve head in the horizontal scans. ONL, outer nuclear layer, highlighted in blue; INL, inner nuclear layer, purple; GCL, ganglion cell layer, orange; and RNFL, retinal nerve fiber layer, arrow. T, temporal; N, nasal; I, inferior, S, superior retina.

Fundus photographs of affected members were taken on certain visits in the era before other imaging modalities were available. A fundus feature of many of the family members was a parafoveal (bull’s eye-like) macular lesion (Krill, 1977). For example, at age 46, PIII-2 had normal-appearing retinal vessels and no peripheral pigmentary changes, but in both maculae, there was a ring of apparent depigmentation surrounding a central area (Figure 3A). Acuities in the two eyes were 20/20 (OD) and 20/70 (OS). A daughter, PIV-2, at age 12, had a parafoveal lesion in each eye and no symptoms. Acuities were OD 20/40 and OS, 20/30. Vessel caliber was normal but there was some depigmentation and sparse bone spicule-like pigment in the superior retina (Figure 3B). PIII-1 at age 47 had features of retinal degeneration throughout the fundus and there were macular pigmentary disturbances (Figure 3C).

Cross-sectional imaging was performed on one of the family members, PIV-3, at age 27 (Figure 3D). Unlike the normal OCT showing typical laminar architecture across the central 50 degrees of retina (upper panels), PIV-3 has severely reduced outer nuclear layer (ONL) thickness in the fovea and across the sampled region (lower panels). There is no foveal bulge (foveal cone outer segments), and acuities are reduced; the outer retinal laminar appearance suggests both rod and cone inner and outer segment losses across the macula (consistent with loss of rod and cone sensitivity noted with chromatic perimetry). Foveal ONL thickness in PIV-3 is 12 μm (defined as average measurement under the foveal pit in horizontal and vertical scans) or 12.1% of the average normal value at this location (98.6 μm, *n* = 15; age range, 8–62 years, SD = 11.1 μm). At a rod rich area (15° superior retina), ONL is 17.3 μm or 31% of average normal value (56.0 μm; *n* = 15; age range, 8–62 years, SD = 6.1 μm).

#### Retina-Wide Rod and Cone Dysfunction by Electroretinography

**FIGURE 4:**
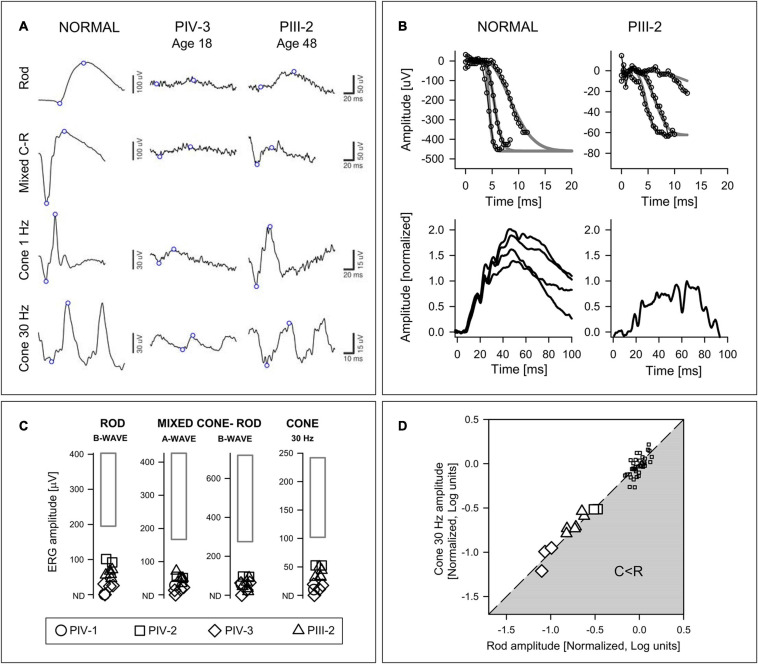
Electroretinography in the *ARL3* family members. **(A)** Rod, mixed (maximum white flash) cone-rod (C-R), and cone ERGs from a subject with normal vision, PIV-3 (age 18) and PIII-2 (age 48). Note the different vertical scales for Normal and Patients. There are larger amplitudes, albeit abnormal, in the father (PIII-2) and the mixed C-R waveform is “negative.” **(B)** P2 and P3 component analyses of rod-isolated ERG photoresponses. (Upper) Rod-isolated a-waves to different stimulus intensities in a normal subject and PIII-2. The smooth curves represent a family of functions describing phototransduction activation best fit to the leading edges of the intensity series. (Lower) Normalized rod P2 components in response to the 3.9 log scot⋅td⋅s intensity stimulus in four normal subjects, and in PIII-2. **(C)** ERG results from four family members compared with normal limits (rectangle: ±2SD from the mean). **(D)** Relationship of rod b-wave amplitude to cone flicker amplitude. Amplitudes are normalized to mean normal values and expressed in log units. Line describes equal reduction of rod and cone amplitudes; gray zone represents greater cone than rod amplitude reduction (C < R). Patient data are larger symbols and small squares are data from normal subjects for comparison.

All family members studied (*n* = 6) had abnormally reduced ERGs (Figure 4C); rod and cone signals in PII-1 and PIII-1 were non-detectable. Waveforms from two members, PIV-3 and PIII-2, separated in age by 30 years illustrate that there was variable expressivity of disease (Figure 4A). PIV-3 at age 18 shows reduced but measurable rod and cone signals. The father, PIII-2, has larger amplitude rod and cone signals but, of interest, the b/a-wave ratio to the maximum white flash that normally produces a mixed cone-rod ERG is reduced (normal mean b/a-wave ratio, 1.6; SD, 0.2; *n* = 96). This “negative ERG” was present in PIII-2 on three recordings (ages 41, 46, and 48).

At age 52 in PIII-2, there was an opportunity to record specialized ERG photoresponses (Figure 4B). The rod component of the mixed rod and cone ERGs to high intensity blue flashes was isolated by the double subtraction technique. The leading edges of the rod-isolated waveforms in a normal subject and in PIII-2 are shown. The phototransduction activation model (gray smooth lines) has been fitted to the responses from an intensity series (small symbols and lines). Normalized rod P2 components from four normal subjects are compared to that of PIII-2; the latter appears smaller than the normal result suggestive of an abnormality at the first synapse of photoreceptors or in rod bipolar cells.

The presence of bull’s eye-like maculopathy in many of the family members and the association of this maculopathy with cone and cone-rod diseases (Thiadens et al., 2012) suggested the value of calculating the relationship between rod and cone ERGs to determine if there was a pattern in these data (Yagasaki and Jacobson, 1989; Figure 4D). The results indicated that dysfunction equally affected rod and cone systems by these ERG parameters, like one of the patterns previously associated with some cone-rod dystrophies (Yagasaki and Jacobson, 1989).

#### Kinetic and Chromatic Static Perimetry

**FIGURE 5:**
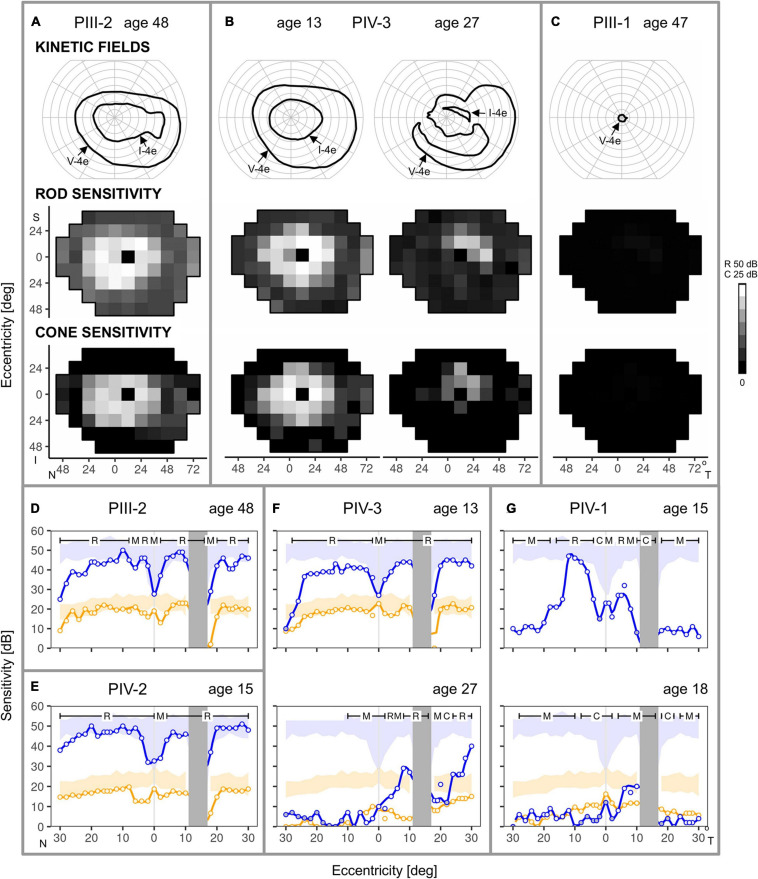
Kinetic and chromatic static perimetry in the family members. **(A)** Full kinetic fields to V-4e but reduced to I-4e test targets in PIII-2 at age 48. Static perimetry shows rod and cone sensitivity is mildly reduced in a large region of central field but there is greater peripheral reduction. **(B)** Serial maps in PIV-3 at age 13 are comparable in pattern to those of PIII-2, but over the subsequent 14 years, there is loss of function in regions of the central field. **(C)** PIII-1, at a similar age to the sibling, PIII-2, has only a small residual central island of vision. **(D–G)** Rod and cone perimetric profiles across the horizontal meridian provide more detail of the central function. **(D,E)** PIII-2 and PIV-2, despite different ages at the visit, show rod and cone sensitivities at the lower limit of normal for most of the central 50°, but there is a decline of function at greater eccentricities. **(F)** PIV-3, at ages 13 and 27, show a sequence of change from central field rod and cone sensitivities at the lower limit of normal to loss of rod and cone function extending from the fovea into the nasal field but some retained peripapillary sensitivities. **(G)** PIV-1 from ages 15–18 shows impaired rod function at the earlier age to barely measurable rod function at the later age. Cone sensitivity was measured at age 18 only and it was abnormally reduced across the horizontal meridian. Blue: 500 nm target, dark-adapted; orange: 600 nm, light-adapted (10 cd.m^–2^ white background); N, nasal; T, temporal; S, superior; I, inferior visual field; R, C, M, rod-, cone- and mixed (rod and cone) -mediated. Shaded areas: normal range (±2SD).

Kinetic and static chromatic fields from three family members illustrate variable expressivity: there are comparable degrees of loss in a father and daughter separated in age by 35 years (PIII-2, age 48 and PIV-3, age 13) and there is also a major difference in severity of the two brothers (PIII-1 and PIII-2) at almost the same age (Figures 5A–C). PIII-2 has a normal extent of field with the V-4e target but a reduced extent with I-4e. In contrast, PIII-1 has just a small central island of vision remaining. Static perimetry maps of sensitivity in PIII-2 and in his daughter, PIV-3 (at ages 48 and 13, respectively), are similar. Peripheral field sensitivity (defined as loci ≥ 24° eccentricity) is far more reduced than the more central field (48° diameter) sensitivity. Both rod- and cone-mediated sensitivity follow the same general pattern across the field. Serial fields in PIV-3 spanning an interval of 14 years in the second and third decades of life show the development of superior, nasal, and inferior mid-peripheral scotomas by age 27; more function is retained temporally. Only rare loci in the periphery had detectable rod function, and the more central (superior-temporal quadrant only) loci had further reduction in sensitivity compared to the results at the earlier age. Cone sensitivity in the periphery was mainly not detectable; centrally, a few loci (superior-temporally) had detectable but reduced function.

Consistent with the results of the larger field sensitivity maps, rod-, and cone-mediated function could be at the lower limit of normal across a relatively wide expanse of the central field (Figures 5D–G). This is illustrated in the dark-adapted two-color horizontal profiles of PIII-2 at age 48 (Figure 5D), and his two daughters (PIV-2 and PIV-3) who are ∼30 years younger (Figures 5E,F). Disease progression in PIV-3 over 14 years (Figure 5F) is notable; nasal field rod function becomes barely detectable, and there is reduction at and near the foveal locus. Patches of rod function remain on both nasal and temporal sides of the optic nerve head. PIV-1, another sister, has greater rod losses at a comparable age to PIV-2 and PIV-3 and progression is notable over the next 3 years. Rod-and mixed-mediated loci lose sufficient rod function to become mostly cone-mediated (Figure 5G).

### Molecular Studies

#### Exome Sequencing Identifies *ARL3* Missense Mutation Segregating With the IRD

Exome sequencing of the proband, PIV-1, identified a total of 573 variants in the 271 RetNet genes. After selecting heterozygous variants consistent with the autosomal dominant pattern observed in the family, we retained 288 variants in 118 genes associated with retinal diseases. After excluding the variants that did not meet our filtering criteria (see section “Materials and Methods”) (Figure 1), we identified three variants: ARL3:NM_004311:exon3:c.A200T:p.D67V, TRPM1:NM_001252020:exon17:c.G2200C:p.A734P, and ALMS1:NM_015120:exon11: c.C9712T:p.R3238C. We deemed the Asp67Val variant in *ARL3* as most likely the causal mutation as this variant was never observed in the normal population and was predicted to be pathogenic across all prediction algorithms (SIFT, PolyPhen2-HDIV, PolyPhen2-HVAR, LRT, MutationTaster, MutationAssessor, FATHMM, MetaSVM, and MetaLR) (Table 2). Additionally, this variant also had high CADD score of 32. The variant in *ALMS1* was predicted to be not pathogenic across multiple predictors and had a low CADD score. Additionally, dominant mutations are not associated with the *ALMS1* and *TRPM1* genes. *ALMS1* mutations cause autosomal recessive Alström syndrome whereas *TRPM1* mutations are associated with recessive congenital stationary night blindness. Thus, we did not pursue these variants for further investigations.

**TABLE 2:** Annotations of p.Asp67Val variant in *ARL3.*

**Feature**	**Prediction**
MAF(ExAC_NFE)	Not observed
CADD score	32
PolyPhen prediction	Damaging
SHIFT prediction	Damaging
LRT prediction	Damaging
MutationTaster prediction	Damaging
MutationAssessor prediction	Harmful
FATHMM prediction	Damaging
PROVEAN prediction	Damaging
MetaSVM prediction	Damaging
MetaLR prediction	Damaging

#### *ARL3* Missense Variant Segregates With the Disease Phenotype

We performed Sanger sequencing to confirm the p.Asp67Val variant in the proband. To further strengthen the evidence of *ARL3* being the causal gene, we obtained samples from five additional family members of which four were affected and one was unaffected. Sanger sequencing analysis showed that the rare heterozygous variant was segregating with the disease phenotype in the family and was absent from the unaffected member (Figure 2B).

#### *ARL3* Missense Variant Is Highly Conserved

**FIGURE 6:**
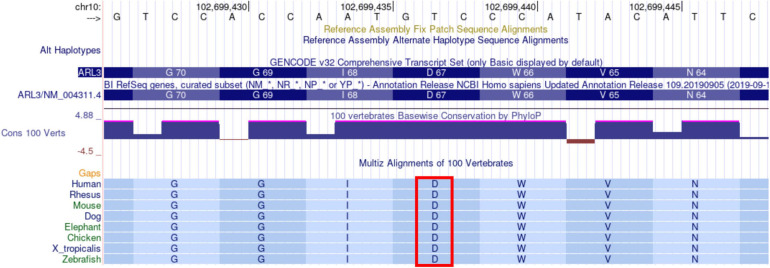
Alignment of ARL3 amino acids surrounding the location corresponding to the D67V mutation in sequences from multiple species. The Asp (D) at residue 67 is indicated by the red rectangle and is conserved across all of the species shown, including human, rhesus, mouse, dog, elephant, chicken, X_tropicalis, and zebrafish. The sequences were aligned with use of the UCSC genome browser.

Multi-species comparisons of genetic variants are a powerful tool for accessing their functional relevance. Thus, we used multi-species alignment from the UCSC genome browser to evaluate the conservation of D67 residue in *ARL3*. The *ARL3* D67 residue is highly conserved across human, rhesus, mouse, dog, elephant, chicken, X_tropicalis, and zebrafish at the amino acid and nucleotide levels (Figure 6). D67 is also conserved in ARL1 and ARL2 (data not shown). Protein coding sequences are highly functionally constrained and thus change very slowly during evolution. The conservation of Asp67 residue across species that diverged from a common ancestor around 40–80 million years ago, such as humans and mice, suggest that this variant is functionally important and mutation/alteration at this position will have an adverse effect on gene function.

#### *ARL3* Missense Variant Disrupts Protein–Protein Interaction

*In silico* analysis of p.Asp67Val predicted a decrease in the ARL3 interactions with three of its interactors, RP2, ARL13B, and UNC119A. All three interactions show decreased affinity as a result of p.Asp67Val substitution (Table 3). We therefore predict that ARL3 mutation will significantly impair ARL3 binding with its known interactors.

**TABLE 3:** Prediction of the protein stability using mCSM-PPI2.

**Protein–protein complex**	**PDB id**	**Organism**	**Predicted Affinity Change (**ΔΔ** G^*Affinity*^) *kcal/mol***	**mCSM-PPI2 prediction**
Arl3-RP2	3BH6	*Mus musculus, Homo sapiens*	−0.534	Decreasing affinity
Arl3GppNHp-UNC119a	4GOJ	*Mus musculus, Homo sapiens*	−0.889	Decreasing affinity
Arl3-Arl13B	5DI3	*Chlamydomonas reinhardtii*	−0.356	Decreasing affinity

#### *ARL3* Missense Variant Is Pathogenic

We used population, computational, and segregation data as evidence in the pathogenicity analysis. Based on the guidelines for classification of pathogenic or likely pathogenic variants by Consensus Recommendation of the American College of Medical Genetics and Genomics and the Association for Molecular Pathology (Richards et al., 2015), the p.Asp67Val variant has “moderate” evidence of pathogenicity based on its absence from control subjects. Evidence of pathogenicity is “supporting” based on co-segregation with the disease in multiple affected family members in a gene known to cause the disease, multiple lines of computational evidence to support a deleterious effect on the gene or gene product (conservation and evolutionary), and patient’s phenotype or family history, which is highly specific for a disease with a single genetic etiology. These observations support the conclusion that p.Asp67Val is pathogenic and causal for the IRD phenotype present in the family.

## Discussion

More than 270 genes have been identified as the cause of IRDs so far (Retinal Information Network, 2021). However, new gene identification has not plateaued for IRDs. A considerable number of yet unknown mutations and IRD genes remain to be identified. Here we report a novel mutation in *ARL3*, which is a ubiquitous small GTPase expressed in ciliated cells of plants and animals and is crucial for ciliogenesis and axoneme formation. The role of *ARL3* in retinal disease was first noticed when it was identified as an RP2 interacting protein (Bartolini et al., 2002; Grayson et al., 2002). Subsequent work showed that mice lacking *Arl3* exhibited photoreceptor degeneration and other ciliary phenotypes affecting renal, hepatic, and pancreatic epithelial tubule structures (Schrick et al., 2006). Homozygous mutation in *ARL3* was identified as a cause of autosomal recessive Joubert syndrome, a neurodevelopmental disorder that may include retinal dystrophy, in two unrelated families (Alkanderi et al., 2018). A role for *ARL3* in non-syndromic autosomal recessive IRDs has also been reported (Sheikh et al., 2019; Fu et al., 2021). Two consanguineous Pakistani families with common ancestry both had affected members with a homozygous c.296 > T (p.Arg99Ile) variant (Sheikh et al., 2019). Novel compound heterozygous *ARL3* variants (c.91A > G, p.Thr31Ala; c.353G > T, p.Cys118Phe) were associated with IRD in a Chinese family (Fu et al., 2021).

The role of *ARL3* in dominant IRDs was first suggested based on the identification of a rare, coding heterozygous p.Tyr90Cys variant in a small family (four available members) of European Caucasian descent (Strom et al., 2016). The same variant was later reported in a Norwegian family of four (two affected and two unaffected) (Holtan et al., 2019). In both families, the variant appeared to be *de novo* in origin as the parents of the proband were homozygous for the wild-type allele. p.Tyr90Cys was predicted to be pathogenic and had low population frequency. Here, we report a different mutation in *ARL3* (p.Asp67Val) segregating in an Ashkenazi Jewish family with a dominant IRD. This variant has never been observed in the normal population and is predicted to be pathogenic across all *in silico* prediction tests. The mutation, p.Asp67Val is located in the ARL3 switch 2 region, which is involved in ARL3 conformation changes upon GDP/GTP exchange (Hanke-Gogokhia et al., 2016). The region between switch 1 and switch 2 is known as the interswitch region, which is involved in the binding of PDEδ, RP2, and UNC119 paralogs (Hanke-Gogokhia et al., 2016). Our *in silico* analyses show that substitution of Asp at position 67 is predicted to disrupt the known interactions of ARL3 with RP2, ARL13B, and UNC119A. Arl13B acts as a guanine nucleotide exchange factor for activation of Arl3 within cilia, and this interaction is needed for the release of intra-ciliary cargos (Gotthardt et al., 2015). ARL3 interaction with its effector Unc119 is critical for the allosteric release of ciliary cargo from UNC119 (Ismail et al., 2012). RP2, on the other hand, functions as a GTPase activating protein specific for ARL3 (Veltel et al., 2008), and disruption of the RP2-ARL3 interaction is likely to lead to dysregulation of the trafficking of the specific kinesin to the cilia tips. It has been also shown that constitutive activation of a dominant form of ARL3 leads to disrupted trafficking of prenylated protein trafficking in rod cells leading to progressive photoreceptor degeneration (Wright et al., 2016, 2018). Thus, we speculate that this mutation is likely to disrupt the GDP/GTP exchange, which could affect the precision at which intra-ciliary cargos are released in the photoreceptor outer segment leading to a retinal degeneration phenotype. Our results present independent genetic evidence of *ARL3* as a cause of dominant IRD.

Is there an *ARL3* retinal phenotype? Prior to the current era of molecular diagnoses, clinicians specializing in retinal degenerations agreed that it was important to subclassify IRD patients based on details of phenotype (Marmor et al., 1983; Pagon, 1988). At disease stages when rod and cone ERGs are detectable, the disorders would be labeled by degree of rod versus cone dysfunction across the retina: rod > cone (RCD, or the more general term RP) or cone > rod (CRD) dystrophies (Marmor et al., 1983). Descriptions of phenotype, however, became less rigorous as more attention was paid to genotype. Patient symptoms of night vision disturbances (nyctalopia) and some extramacular pigmentary retinopathy by funduscopy or fundus photography tended to qualify as RP, while reduced visual acuity and macular pigmentary change without other symptoms became a diagnosis of CRD. When evidence is presented for pathogenicity of a gene mutation, the clinical nomenclature for the disease becomes incorporated into various databases. Full circle has been reached now that decisions need to be made about which patients with what disease features are candidates for a gene-based clinical trial. The gene diagnosis is obviously the key inclusion criterion but we are not at such an advanced state of therapeutics that a molecular diagnosis is all we need to proceed to a clinical trial. A clinical diagnosis and some understanding of the disease phenotype and its natural history are helpful to determine the feasibility or value (to the patient) of a trial and design its outcomes; and to decide if an animal model used for preclinical work is a faithful mimic of the human disease.

The affected family members with dominant *ARL3* p.Asp67Val in the current study manifested equal rod and cone dysfunction by ERG (Figure 4D; Yagasaki and Jacobson, 1989), maculopathy in the form of bull’s eye changes even at early stages, and late stage loss of macular and peripheral vision with retina-wide pigmentary retinopathy. Bull’s eye maculopathy is not specific and has been associated with toxic disorders as well as IRDs but especially cone dystrophy and CRD (Krill, 1977; Michaelides et al., 2007; Thiadens et al., 2012). One family member had a negative ERG, suggesting inner retinal dysfunction (Audo et al., 2008); whether this human phenotypic feature relates to the finding of abnormal rod cell migration in an ARL3 murine mutant requires further study (Wright et al., 2016). Rather than tabulating or trying to describe similarities and differences between the ARL3 phenotype and the extensive list of genetically defined diseases now known to cause IRDs (e.g., see Verbakel et al., 2018; Table 2), we compared disease features with those previously reported as associated with *ARL3* mutations. In one dominant family with the p.Tyr90Cys *ARL3* variant (Strom et al., 2016), the mother at age 58 years had modest acuity reduction (20/60 and 20/80). Fundi were described as having “classic” midperipheral pigmentation. A 27-year-old daughter had reduced visual acuities of 20/80 and 20/60. Fundi were said to have “bone-spicules” in the periphery. A 30-year-old son had near normal acuities (20/20, 20/25) and cystoid macular edema. Both siblings were said to have peripheral vision losses. No ERG results or fundus images were shown. The disease was discussed as RP but the main focus of the report was the association of this IRD with the *ARL3* variant. The limited phenotypic data make it difficult to compare these 3 patients with those of the family we report herein.

In the second dominant p.Tyr90Cys family, an affected male member recalled nyctalopia in the first decade of life and 20/80 acuities at age 38, further reducing to 20/125, 20/200 at age 52. The ERG at age 39 was reported as not detectable. Retina-wide pigmentary retinopathy was present at age 57. Central atrophy was present by multiple imaging modalities. The affected son had modestly reduced acuities at age 16 (20/32 and 20/50); a rod ERG was not detectable and cone ERGs were reported to be reduced. Pigmentary retinopathy was shown on fundus photographs, and there was central atrophy by OCT and autofluorescence imaging. Macular atrophy in both family members at early and later stages accompanies the retina-wide pigmentary retinopathy. This is neither “typical RP” nor clearly CRD. The disease expression with peripheral retinal degeneration and maculopathy is interestingly similar to that in *ARL3* family members of the current study.

Autosomal recessive IRDs caused by *ARL3* mutations have also been described by phenotype (Sheikh et al., 2019; Fu et al., 2021). In two Pakistani families that shared the same homozygous pathogenic *ARL3* variant (p.Arg99Ile; Sheikh et al., 2019), there was imaging evidence of maculopathy (whether atrophy or bull’s eye changes) in the second to the fourth decades of life and reduced acuities. Visual fields are reported as not showing peripheral vision loss, but no details are provided. It is not clear if color vision is affected—textual reference is made to it being normal but a table indicates abnormalities in some patients. ERGs, albeit using skin electrodes leading to a limited range of amplitude (Bradshaw et al., 2004), had a rod and cone signal relationship that suggested cone > rod dysfunction, unlike the equal cone and rod ERG dysfunction in the present study. From the information provided, the disease expression due to this homozygous *ARL3* variant may be that of a CRD or a maculopathy in these myopic patients. The proband of the Chinese family with a compound *ARL3* heterozygote (p.Thr31Ala, p.Cys118Phe; age 19, male) had reduced acuities and color vision abnormalities. Visual fields are reported as a “tunnel” but only midperipheral fields are shown: a superior temporal abnormality corresponds to the pigmentary changes in the inferior nasal retina. The ERGs are not readily interpretable as shown (without normal data for reference) but are said to be reduced for rod and cone responses. OCTs have abnormalities in outer retinal layers, but there appears to be a well-preserved outer nuclear layer in the scans. The heterozygous father (p.Thr31Ala), the only other family member with a phenotype, is described as having normal visual acuity, a central scotoma, color vision abnormalities, OCT with foveal area preservation but para- and peri-central outer retinal layer abnormalities, and possibly a bull’s eye maculopathy (“circular degeneration around macular fovea”; Fu et al., 2021). Both family members seem to show a widespread rod and cone degeneration with different severities.

A conclusion from our report taken together with the other published data of *ARL3* disease is that both rod and cone photoreceptors are affected across the retina and maculopathy tends to be present even at early stages. Forcing the phenotype into a simplified binary clinical classification of RCD (RP) versus CRD may only lead to another molecular disease being labeled as showing phenotypic variability. A more general description for now should open the door for more detailed observations in future patients identified with non-syndromic *ARL3* mutations.

## Data Availability Statement

The datasets presented in this study can be found in online repositories. The names of the repository/repositories and accession number(s) can be found below: https://databases.lovd.nl/shared/individuals/00375519.

## Ethics Statement

The studies involving human participants were reviewed and approved by the University of Pennsylvania. Written informed consent to participate in this study was provided by the participants or the participants’ legal guardian/next of kin. Written informed consent was obtained from the individual(s), and minor(s)’ legal guardian/next of kin, for the publication of any potentially identifiable images or data included in this article.

## Author Contributions

SJ and AC designed and supervised the clinical aspects of the study. RR, ME, and ASw performed and interpreted the molecular results. AR, ASu, and RS analyzed and managed the data from phenotyping. All authors contributed to the article and approved the submitted version.

## Conflict of Interest

The authors declare that the research was conducted in the absence of any commercial or financial relationships that could be construed as a potential conflict of interest.

## Publisher’s Note

All claims expressed in this article are solely those of the authors and do not necessarily represent those of their affiliated organizations, or those of the publisher, the editors and the reviewers. Any product that may be evaluated in this article, or claim that may be made by its manufacturer, is not guaranteed or endorsed by the publisher.

## Footnotes

1
https://sph.uth.edu/retnet/

2
http://picard.sourceforge.net

3
https://sph.uth.edu/Retnet/sum-dis.htm

4
http://genome.ucsc.edu

## Supplementary Material

Supplementary Figure 1 **(A)** Visual acuity as a function of age in five members of the *ARL3* family. Longitudinal data are shown as symbols connected by lines. **(B)** Visual acuities in four of the patients replotted as a function of years after an estimated onset of decline in acuity. The dashed line corresponds to the group decline in acuity (0.029 logMAR/year or 6.9% per year; *n* = 8 eyes, mixed-effects model).

## References

[B1] Aleman T. S. Soumittra N. Cideciyan A. V. Sumaroka A. M. Ramprasad V. L. Herrera W. (2009). CERKL mutations cause an autosomal recessive cone-rod dystrophy with inner retinopathy. *Invest. Ophthalmol. Vis. Sci.* 50 5944–5954. 10.1167/iovs.09-3982 19578027

[B2] Alkanderi S. Molinari E. Shaheen R. Elmaghloob Y. Stephen L. A. Sammut V. (2018). ARL3 mutations cause joubert syndrome by disrupting ciliary protein composition. *Am. J. Hum. Genet.* 103 612–620. 10.1016/j.ajhg.2018.08.015 30269812 PMC6174286

[B3] Audo I. Robson A. G. Holder G. E. Moore A. T. (2008). The negative ERG: clinical phenotypes and disease mechanisms of inner retinal dysfunction. *Surv. Ophthalmol.* 53, 16–40. 10.1016/j.survophthal.2007.10.010 18191655

[B4] Azari A. A. Aleman T. S. Cideciyan A. V. Schwartz S. B. Windsor E. A. Sumaroka A. (2006). Retinal disease expression in bardet-biedl syndrome-1 (BBS1) is a spectrum from maculopathy to retina-wide degeneration. *Invest. Ophthalmol. Vis. Sci.* 47 5004–5010. 10.1167/iovs.06-0517 17065520

[B5] Bartolini F. Bhamidipati A. Thomas S. Schwahn U. Lewis S. A. Cowan N. J. (2002). Functional overlap between retinitis pigmentosa 2 protein and the tubulin-specific chaperone cofactor C. *J. Biol. Chem.* 277 14629–14634. 10.1074/jbc.M200128200 11847227

[B6] Beryozkin A. Shevah E. Kimchi A. Mizrahi-Meissonnier L. Khateb S. Ratnapriya R. (2015). Whole exome sequencing reveals mutations in known retinal disease genes in 33 out of 68 Israeli families with inherited retinopathies. *Sci. Rep.* 5:13187. 10.1038/srep13187 26306921 PMC4549705

[B7] Birtel J. Gliem M. Mangold E. Müller P. L. Holz F. G. Neuhaus C. (2018). Next-generation sequencing identifies unexpected genotype-phenotype correlations in patients with retinitis pigmentosa. *PloS One* 13:e0207958. 10.1371/journal.pone.0207958 30543658 PMC6292620

[B8] Bolger A. M. Lohse M. Usadel B. (2014). Trimmomatic: a flexible trimmer for illumina sequence data. *Bioinformatics* 30 2114–2120. 10.1093/bioinformatics/btu170 24695404 PMC4103590

[B9] Booij J. C. Bakker A. Kulumbetova J. Moutaoukil Y. Smeets B. Verheij J. (2011). Simultaneous mutation detection in 90 retinal disease genes in multiple patients using a custom-designed 300-kb retinal resequencing chip. *Ophthalmology* 118 160–167. 10.1016/j.ophtha.2010.04.022 20801516

[B10] Bradshaw K. Hansen R. Fulton A. (2004). Comparison of ERGs recorded with skin and corneal-contact electrodes in normal children and adults. *Doc. Ophthalmol.* 109 43–55. 10.1007/s10633-004-1751-3 15675199

[B11] Bramall A. N. Wright A. F. Jacobson S. G. McInnes R. R. (2010). The genomic, biochemical, and cellular responses of the retina in inherited photoreceptor degenerations and prospects for the treatment of these disorders. *Ann. Rev. Neurosci.* 33 441–472. 10.1146/annurev-neuro-060909-153227 20572772

[B12] Cideciyan A. V. (2000). In vivo assessment of photoreceptor function in human diseases caused by photoreceptor-specific gene mutations. *Methods Enzymol.* 316 611–626. 10.1016/s0076-6879(00)16753-9 10800705

[B13] Cideciyan A. V. Jacobson S. G. (1993). Negative electroretinograms in retinitis pigmentosa. *Invest. Ophthalmol. Vis. Sci.* 34 3253–3263. 8225860

[B14] Cideciyan A. V. Jacobson S. G. (1996). An alternative phototransduction model for human rod and cone ERG a-waves: normal parameters and variation with age. *Vision Res.* 36 2609–2621. 10.1016/0042-6989(95)00327-4 8917821

[B15] DePristo M. A. Banks E. Poplin R. Garimella K. V. Maguire J. R. Hartl C. (2011). A framework for variation discovery and genotyping using next-generation DNA sequencing data. *Nat. Genet.* 43 491–498. 10.1038/ng.806 21478889 PMC3083463

[B16] Evans R. J. Schwarz N. Nagel-Wolfrum K. Wolfrum U. Hardcastle A. J. Cheetham M. E. (2010). The retinitis pigmentosa protein RP2 links pericentriolar vesicle transport between the Golgi and the primary cilium. *Hum. Mol. Genet.* 19 1358–1367. 10.1093/hmg/ddq012 20106869

[B17] Fahim A. T. Daiger S. P. Weleber R. G. (2000). “Nonsyndromic retinitis pigmentosa overview,” in *GeneReviews*, eds Adam M. P. Ardinger H. H. Pagon R. A. Wallace S. E. Bean L. J. H. Mirzaa G. (Seattle, WA: University of Washington). 20301590

[B18] Frederick J. M. Hanke-Gogokhia C. Ying G. Baehr W. (2020). Diffuse or hitch a ride: how photoreceptor lipidated proteins get from here to there. *Biol. Chem.* 401 573–584. 10.1515/hsz-2019-0375 31811799

[B19] Fu L. Li Y. Yao S. Guo Q. You Y. Zhu X. (2021). Autosomal recessive rod-cone dystrophy associated with compound heterozygous variants in ARL3 gene. *Front. Cell Dev. Biol.* 9:635424. 10.3389/fcell.2021.635424 33748123 PMC7969994

[B20] Garafalo A. V. Cideciyan A. V. Héon E. Sheplock R. Pearson A. WeiYang Yu C. (2020). Progress in treating inherited retinal diseases: early subretinal gene therapy clinical trials and candidates for future initiatives. *Prog. Retin. Eye Res.* 77:100827. 10.1016/j.preteyeres.2019.100827 31899291 PMC8714059

[B21] Gotthardt K. Lokaj M. Koerner C. Falk N. Gießl A. Wittinghofer A. (2015). A G-protein activation cascade from Arl13B to Arl3 and implications for ciliary targeting of lipidated proteins. *Elife* 4:e11859. 10.7554/eLife.11859 26551564 PMC4868535

[B22] Grayson C. Bartolini F. Chapple J. P. Willison K. R. Bhamidipati A. Lewis S. A. (2002). Localization in the human retina of the X-linked retinitis pigmentosa protein RP2, its homologue cofactor C and the RP2 interacting protein Arl3. *Hum. Mol. Genet.* 11 3065–3074. 10.1093/hmg/11.24.3065 12417528

[B23] Hanke-Gogokhia C. Wu Z. Gerstner C. D. Frederick J. M. Zhang H. Baehr W. (2016). Arf-like protein 3 (ARL3) regulates protein trafficking and ciliogenesis in mouse photoreceptors. *J. Biol. Chem.* 291 7142–7155. 10.1074/jbc.M115.710954 26814127 PMC4807295

[B24] Hejtmancik J. F. Daiger S. P. (2020). Understanding the genetic architecture of human retinal degenerations. *Proc. Natl. Acad. Sci. U.S.A.* 117 3904–3906. 10.1073/pnas.1922925117 32034100 PMC7049104

[B25] Holtan J. P. Teigen K. Aukrust I. Bragadóttir R. Houge G. (2019). Dominant ARL3-related retinitis pigmentosa. *Ophthalmic Genet.* 40 124–128. 10.1080/13816810.2019.1586965 30932721

[B26] Ismail S. Chen Y.-X. Miertzschke M. Vetter I. R. Koerner C. Wittinghofer A. (2012). Structural basis for Arl3-specific release of myristoylated ciliary cargo from UNC119. *EMBO J*. 31 4085–4094. 10.1038/emboj.2012.257 22960633 PMC3474929

[B27] Jacobson S. G. Cideciyan A. V. Aleman T. S. Pianta M. J. Sumaroka A. Schwartz S. B. (2003). Crumbs homolog 1 (CRB1) mutations result in a thick human retina with abnormal lamination. *Hum. Mol. Genet.* 12 1073–1078. 10.1093/hmg/ddg117 12700176

[B28] Jacobson S. G. Voigt W. J. Parel J. M. Apáthy P. P. Nghiem-Phu L. Myers S. W. (1986). Automated light- and dark-adapted perimetry for evaluating retinitis pigmentosa. *Ophthalmology* 93 1604–1611. 10.1016/s0161-6420(86)33522-x 3808619

[B29] Jacobson S. G. Yagasaki K. Feuer W. J. Roman A. J. (1989). Interocular asymmetry of visual function in heterozygotes of X-linked retinitis pigmentosa. *Exp. Eye Res.* 48 679–691. 10.1016/0014-4835(89)90009-2 2737262

[B30] Krill A. E. (1977). *Krill’s Hereditary Retinal and Choroidal Diseases*, Vol. 2. Hagerstown, MD: Harper.

[B31] Kruczek K. Qu Z. Gentry J. Fadl B. R. Gieser L. Hiriyanna S. (2021). Gene therapy of dominant CRX-Leber congenital amaurosis using patient stem cell-derived retinal organoids. *Stem Cell Rep.* 16 252–263. 10.1016/j.stemcr.2020.12.018 33513359 PMC7878833

[B32] Kruczek K. Swaroop A. (2020). Pluripotent stem cell-derived retinal organoids for disease modeling and development of therapies. *Stem Cells* 38 1206–1215. 10.1002/stem.3239 32506758 PMC7586922

[B33] Marmor M. F. Aguirre G. Arden G. Berson E. Birch D. G. Boughman J. A. (1983). Retinitis pigmentosa: a symposium on terminology and methods of examination. *Ophthalmology* 90 126–131. 6856249

[B34] Michaelides M. Chen L. L. Brantley M. A. Jr. Andorf J. L. Isaak E. M. Jenkins S. A. (2007). ABCA4 mutations and discordant ABCA4 alleles in patients and siblings with bull’s-eye maculopathy. *Br. J. Ophthalmol.* 91 1650–1655. 10.1136/bjo.2007.118356 18024811 PMC2095527

[B35] O’Sullivan J. Mullaney B. G. Bhaskar S. S. Dickerson J. E. Hall G. O’Grady A. (2012). A paradigm shift in the delivery of services for diagnosis of inherited retinal disease. *J. Med. Genet.* 49 322–326. 10.1136/jmedgenet-2012-100847 22581970

[B36] Pagon R. A. (1988). Retinitis pigmentosa. *Surv. Ophthalmol.* 33 133–177. 10.1016/0039-6257(88)90085-9 3068820

[B37] Ratnapriya R. Swaroop A. (2013). Genetic architecture of retinal and macular degenerative diseases: the promise and challenges of next-generation sequencing. *Genome Med.* 5:84. 24112618 10.1186/gm488 PMC4066589

[B38] Retinal Information Network (2021). *RetNet, the Retinal Information Network.* Available online at: https://sph.uth.edu/RetNet/ (Accessed July 1, 2020).

[B39] Richards S. Aziz N. Bale S. Bick D. Das S. Gastier-Foster J. (2015). Standards and guidelines for the interpretation of sequence variants: a joint consensus recommendation of the american college of medical genetics and genomics and the association for molecular pathology. *Genet. Med*. 17 405–424. 10.1038/gim.2015.30 25741868 PMC4544753

[B40] Roberts L. Ratnapriya R. du Plessis M. Chaitankar V. Ramesar R. S. Swaroop A. (2016). Molecular diagnosis of inherited retinal diseases in indigenous African populations by whole-exome sequencing. *Invest. Ophthalmol. Vis. Sci.* 57 6374–6381. 10.1167/iovs.16-19785 27898983 PMC5132076

[B41] Rodrigues C. H. M. Myung Y. Pires D. E. V. Ascher D. B. (2019). MCSM-PPI2: predicting the effects of mutations on protein-protein interactions. *Nucleic Acids Res*. 47 W338–W344. 10.1093/nar/gkz383 31114883 PMC6602427

[B42] Roman A. J. Schwartz S. B. Aleman T. S. Cideciyan A. V. Chico J. D. Windsor E. A. (2005). Quantifying rod photoreceptor-mediated vision in retinal degenerations: dark-adapted thresholds as outcome measures. *Exp. Eye Res.* 80 259–272. 10.1016/j.exer.2004.09.00 15670804

[B43] Sánchez-Bellver L. Toulis V. Marfany G. (2021). On the wrong track: alterations of ciliary transport in inherited retinal dystrophies. *Front. Cell Dev. Biol.* 9:623734. 10.3389/fcell.2021.623734 33748110 PMC7973215

[B44] Schrick J. J. Vogel P. Abuin A. Hampton B. Rice D. S. (2006). ADP-ribosylation factor-like 3 is involved in kidney and photoreceptor development. *Am. J. Pathol.* 168 1288–1298. 10.2353/ajpath.2006.050941 16565502 PMC1606550

[B45] Schwarz N. Hardcastle A. J. Cheetham M. E. (2012). Arl3 and RP2 mediated assembly and traffic of membrane associated cilia proteins. *Vision Res.* 75 2–4. 10.1016/j.visres.2012.07.016 22884633

[B46] Sheikh S. A. Sisk R. A. Schiavon C. R. Waryah Y. M. Usmani M. A. Steel D. H. (2019). Homozygous variant in ARL3 causes autosomal recessive cone rod dystrophy. *Invest. Ophthalmol. Vis. Sci.* 60 4811–4819. 10.1167/iovs.19-27263 31743939 PMC6944245

[B47] Strom S. P. Clark M. J. Martinez A. Garcia S. Abelazeem A. A. Matynia A. (2016). De novo occurrence of a variant in ARL3 and apparent autosomal dominant transmission of retinitis pigmentosa. *PLoS One* 11:e0150944. 10.1371/journal.pone.0150944 26964041 PMC4786330

[B48] Sudharsan R. Beltran W. A. (2019). Progress in gene therapy for rhodopsin autosomal dominant retinitis pigmentosa. *Adv. Exp. Med. Biol.* 1185 113–118. 10.1007/978-3-030-27378-1_19 31884598 PMC7217593

[B49] Sumaroka A. Cideciyan A. V. Charng J. Wu V. Powers C. A. Iyer B. S. (2019). Autosomal dominant retinitis pigmentosa due to class B *Rhodopsin* mutations: an objective outcome for future treatment trials. *Int. J. Mol. Sci.* 20:5344. 10.3390/ijms20215344 31717845 PMC6861901

[B50] Thiadens A. A. Phan T. M. Zekveld-Vroon R. C. Leroy B. P. van den Born L. I. Hoyng C. B. (2012). Clinical course, genetic etiology, and visual outcome in cone and cone-rod dystrophy. *Ophthalmology* 119 819–826. 10.1016/j.ophtha.2011.10.011 22264887

[B51] Veltel S. Gasper R. Eisenacher E. Wittinghofer A. (2008). The retinitis pigmentosa 2 gene product is a GTPase-activating protein for arf-like 3. *Nat. Struct. Mol. Biol.* 15 373–380. 10.1038/nsmb.1396 18376416

[B52] Verbakel S. K. van Huet R. Boon C. den Hollander A. I. Collin R. Klaver C. (2018). Non-syndromic retinitis pigmentosa. *Prog. Retin. Eye Res.* 66 157–186. 10.1016/j.preteyeres.2018.03.005 29597005

[B53] Wang K. Li M. Hakonarson H. (2010). ANNOVAR: functional annotation of genetic variants from high-throughput sequencing data. *Nucleic Acids Res.* 38:e164. 10.1093/nar/gkq603 20601685 PMC2938201

[B54] Wright A. F. Chakarova C. F. Abd El-Aziz M. M. Bhattacharya S. S. (2010). Photoreceptor degeneration: genetic and mechanistic dissection of a complex trait. *Nat. Rev. Genet.* 11 273–284. 10.1038/nrg2717 20212494

[B55] Wright Z. C. Loskutov Y. Murphy D. Stoilov P. Pugacheva E. Goldberg A. F. X. (2018). ADP-ribosylation factor-like 2 (ARL2) regulates cilia stability and development of outer segments in rod photoreceptor neurons. *Sci. Rep.* 8:16967. 30446707 10.1038/s41598-018-35395-3 PMC6240099

[B56] Wright Z. C. Singh R. K. Alpino R. Goldberg A. F. Sokolov M. Ramamurthy V. (2016). ARL3 regulates trafficking of prenylated phototransduction proteins to the rod outer segment. *Hum. Mol. Genet*. 25 2031–2044. 10.1093/hmg/ddw077 26936825 PMC5062590

[B57] Yagasaki K. Jacobson S. G. (1989). Cone-rod dystrophy. phenotypic diversity by retinal function testing. *Arch. Ophthalmol.* 107 701–708. 10.1001/archopht.1989.01070010719034 2719580

