# Supplementary figures and images for: A Novel ARL3 Gene Mutation Associated With Autosomal Dominant Retinal Degeneration
Source: Front Cell Dev Biol. 2021 Aug 17;9:720782. doi: 10.3389/fcell.2021.720782 (PMC8416110; doi:10.3389/fcell.2021.720782)

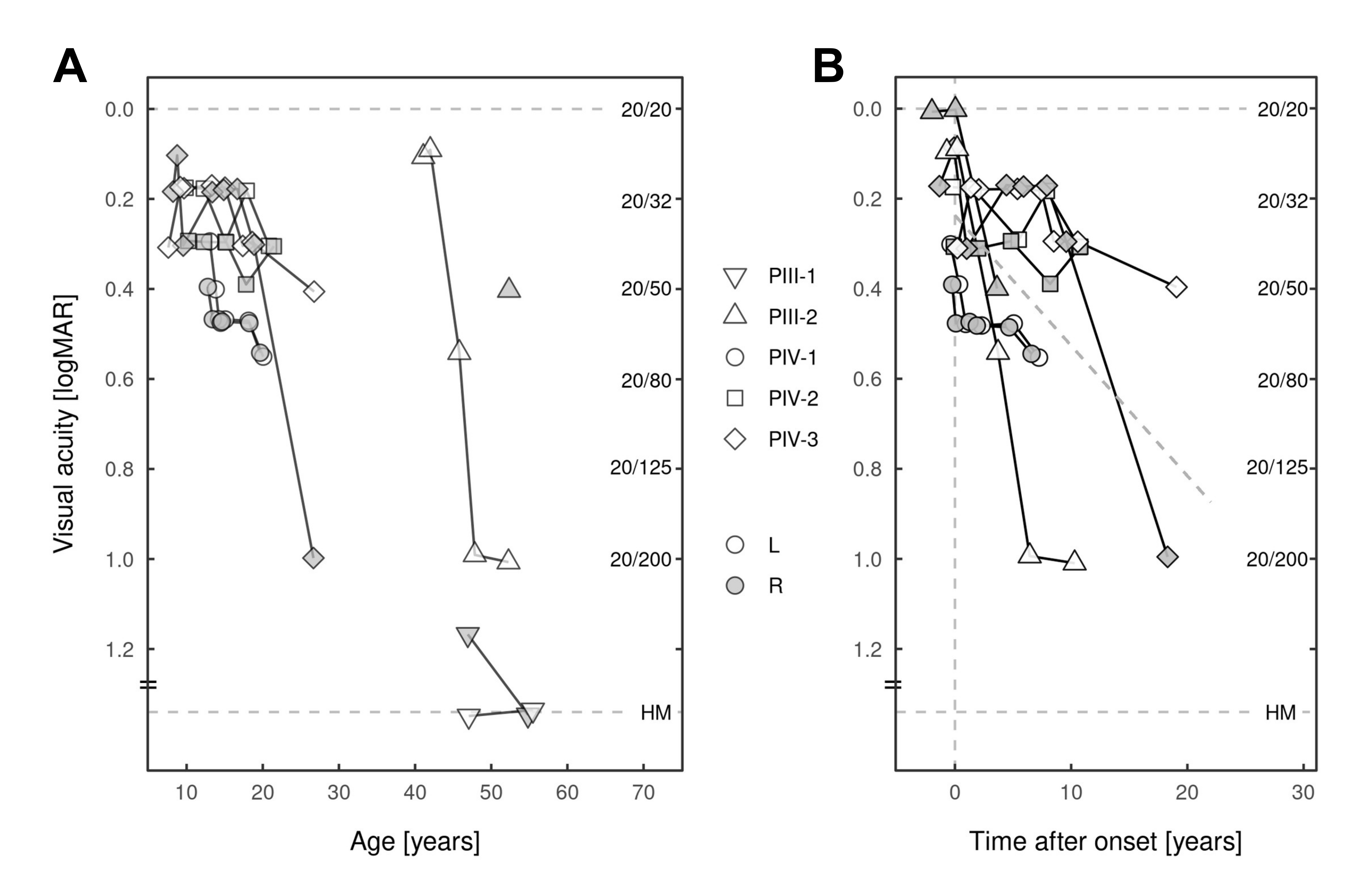

Supplement: Supplementary Figure 1 — (A) Visual acuity as a function of age in five members of the ARL3 family. Longitudinal data are shown as symbols connected by lines. (B) Visual acuities in four of the patients replotted as a function of years after an estimated onset of decline in acuity. The dashed line corresponds to the group decline in acuity (0.029 logMAR/year or 6.9% per year; n = 8 eyes, mixed-effects model). [file Image_1.TIF]
